# Extrusion of Neutrophil Extracellular Traps (NETs) Negatively Impacts Canine Sperm Functions: Implications in Reproductive Failure

**DOI:** 10.3390/ijms25116216

**Published:** 2024-06-05

**Authors:** Marion León, Claudia Moya, Rodrigo Rivera-Concha, Felipe Pezo, Pamela Uribe, Mabel Schulz, Raúl Sánchez, Anja Taubert, Carlos Hermosilla, Fabiola Zambrano

**Affiliations:** 1 Center of Excellence in Translational Medicine—Scientific and Technological Bioresource Nucleus (CEMT-BIOREN), Faculty of Medicine, Universidad de La Frontera, Temuco 4780000, Chile; m.leon06@ufromail.cl (M.L.); c.moya03@ufromail.cl (C.M.); r.rivera07@ufromail.cl (R.R.-C.); felipe.pezo@ufrontera.cl (F.P.); pamela.uribe@ufrontera.cl (P.U.); mabel.schulz@ufrontera.cl (M.S.); raul.sanchez@ufrontera.cl (R.S.); 2 Ph.D. Program in Medical Sciences, Faculty of Medicine, Universidad de La Frontera, Temuco 4780000, Chile; 3 Department of Internal Medicine, Faculty of Medicine, Universidad de La Frontera, Temuco 4780000, Chile; 4 Department of Preclinical Sciences, Faculty of Medicine, Universidad de La Frontera, Temuco 4780000, Chile; 5 Institute of Parasitology, Justus Liebig University Giessen, 35392 Giessen, Germany; anja.taubert@vetmed.uni-giessen.de (A.T.); carlos.r.hermosilla@vetmed.uni-giessen.de (C.H.)

**Keywords:** canine, spermatozoon, PMNs, neutrophil extracellular traps (NETs), sperm functionality

## Abstract

Reproductive failure in dogs is often due to unknown causes, and correct diagnosis and treatment are not always achieved. This condition is associated with various congenital and acquired etiologies that develop inflammatory processes, causing an increase in the number of leukocytes within the female reproductive tract (FRT). An encounter between polymorphonuclear neutrophils (PMNs) and infectious agents or inflammation in the FRT could trigger neutrophil extracellular traps (NETs), which are associated with significantly decreased motility and damage to sperm functional parameters in other species, including humans. This study describes the interaction between canine PMNs and spermatozoa and characterizes the release of NETs, in addition to evaluating the consequences of these structures on canine sperm function. To identify and visualize NETs, May–Grünwald Giemsa staining and immunofluorescence for neutrophil elastase (NE) were performed on canine semen samples and sperm/PMN co-cultures. Sperm viability was assessed using SYBR/PI and acrosome integrity was assessed using PNA-FITC/PI by flow cytometry. The results demonstrate NETs release in native semen samples and PMN/sperm co-cultures. In addition, NETs negatively affect canine sperm function parameters. This is the first report on the ability of NETs to efficiently entrap canine spermatozoa, and to provide additional data on the adverse effects of NETs on male gametes. Therefore, NETs formation should be considered in future studies of canine reproductive failure, as these extracellular fibers and NET-derived pro-inflammatory capacities will impede proper oocyte fertilization and embryo implantation. These data will serve as a basis to explain certain reproductive failures of dogs and provide new information about triggers and molecules involved in adverse effects of NETosis for domestic pet animals.

## 1. Introduction

Reproductive failure in dogs is increasing in clinical practice and is of paramount importance to breeders and kennel clubs worldwide. Proper mating to maintain pure breeds seems essential, and the same goes for dogs that have won several national/international dog shows or rare dog breeds (e.g., Azawakh, Bergamasco Sheepdog, Lagotto Romagnolo, Otterhound, and Patagonian Sheepdog) that have been genetically selected for several generations [1]. The underlying cause of reproductive failure in dogs is unknown in most cases. However, it has been observed that approximately 10% of dogs that receive an accurate diagnosis and appropriate treatment can have their fertility restored [2]. The possible causes of reproductive failure have been grouped into congenital infertility and acquired infertility. The causes are associated with factors such as prostate and testicular problems, hormonal changes, infectious diseases, and certain medications [3,4]. They may also be associated with lower total antioxidant capacity and increased protein peroxidation in seminal plasma due to oxidative stress [5]. In females, the causes of reproductive failure may be hormonal factors, cystic endometrial hyperplasia (CEH), endometrial gland inflammation, or endometrial atrophy [6]. The female reproductive tract (FRT) contains several aerobic bacterial populations in the vaginal area and uterus; however, infectious agents leading to vaginitis, cervicitis, and uterine pathology can also contribute to infertility in the canine female [7]. 

Polymorphonuclear neutrophils (PMNs) are the most abundant leukocyte population in the blood and are essential effector cells of the innate immune system [8]. In response to inflammatory stimuli, PMNs migrate from the circulating blood to the site of infection and eliminate microorganisms through phagocytosis, degranulation, and NETosis [9,10]. NETosis was first described against bacteria in which PMNs generate neutrophil extracellular traps (NETs), structures composed of granules and nuclear components that disarm and kill bacteria extracellularly [8,9]. NET formation is an active process involving rearrangement of the nuclear and granular architecture, which is released after cell membrane rupture and cell death [8,11,12]. The cell death process is dependent on the NADPH oxidase complex and subsequent activation of the PAD4 protein, which leads to the extrusion of a mixture of nuclear DNA and cytoplasmic granule content, resulting in the formation of extracellular DNA network structures as its main component. In addition, histones (H1, H2A/H2B, H3, H4) and proteins such as neutrophil elastase (NE), myeloperoxidase (MPO), cathepsin G, lactoferrin, and gelatinase are also formed [8,9,12]. Although PMNs infiltration into the FRT is thought to be responsible for maintaining a healthy vaginal environment, the physiological role of PMNs and NETosis in vaginal discharge remains to be elucidated [13,14]. The site of sperm deposition varies between species, which may influence the extent of leukocyte infiltration into different areas of the FRT and their accumulation at different sites [15]. Inflammatory processes and bacteria induce leukocyte infiltration, with PMNs being the predominant cells of the white line observed in vaginal smears during the proestrus and estrus phases of the canine cycle [14]. In addition, there is a relationship with the increase in leukocytes, depending on the bitch’s estrous cycle stage [14,16,17]. 

Since the discovery of NETs and their central role against invasive pathogens in 2004, no studies have been conducted on the role NETosis might play in the canine reproductive system. Possible triggers and/or adverse effects of uncontrolled NETs release within the FRT have also not been investigated. Therefore, this study investigated the interaction of NETs with canine spermatozoa and assessed the consequences for male gametes after the NETosis process.

## 2. Results

### 2.1. Visualization of NETs Induced by Dog Sperm Using Immunofluorescence and Optical Microscopy

**Figure 1:**
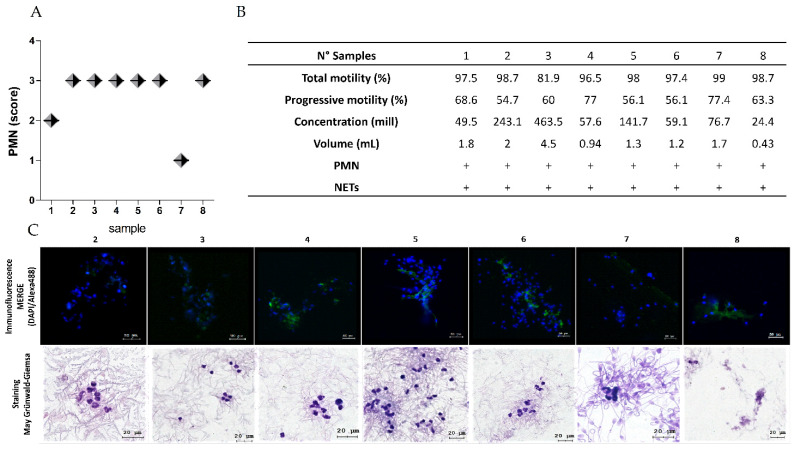
Characterization of dog semen samples. The chart in (**A**) shows the score determined for the presence of PMNs in each sperm sample, with 1 being scarce, 2 moderate, and 3 abundant. The table in (**B**) shows the results of the quality parameters of the semen sample. The images in (**C**) are representative of fluorescence microscopy and light microscopy observed with the 20× and 100× objective, respectively, showing PMNs infiltration and NET production (scale bars = 20 μm).

**Figure 2:**
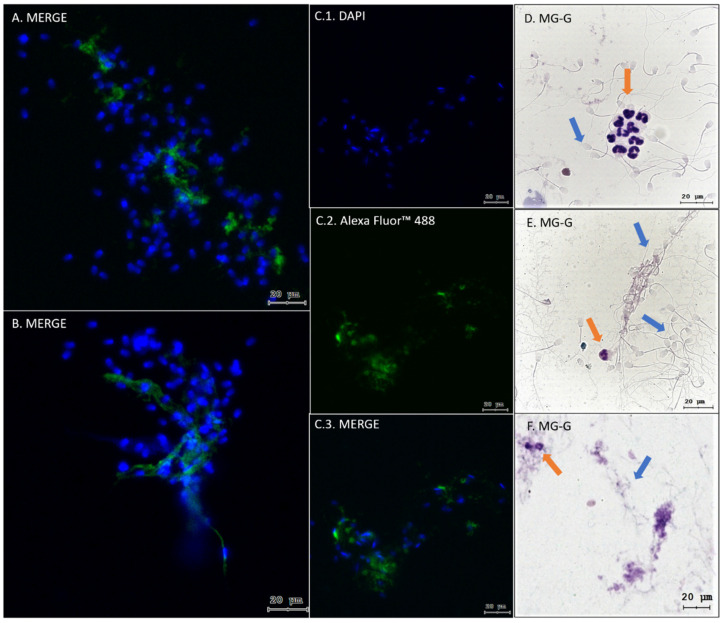
Representative immunofluorescence images show PMNs releasing NETs, and entrapped sperm present in dog semen samples by immunofluorescence. (**A**,**B**,**C.3**) MERGE indicates the co-location of both channels (DAPI and Alexa Fluor™ 488). (**C.1**) DAPI marker with blue fluorescence indicates nuclear sperm DNA and PMNs. (**C.2**) Alexa Fluor™ 488 marker with green fluorescence indicates NE from released NETs. (**D**–**F**) contains representative images of the May–Grünwald Giemsa stain, where the orange arrows indicate PMNs present in the samples and the blue arrows indicate sperm (scale bars = 20 μm).

The quantification analysis by optical microscopy revealed a significant influx of PMNs in all the dog semen samples (n = 8), where most had a PMNs score equal to 3, i.e., abundant according to the above-mentioned criteria (Figure 1A,B), in addition to visualizing structures similar to the NETs through simple May–Grünwald Giemsa staining (Figure 1C and Figure 2D–F), which was corroborated using immunofluorescence. The presence of NE in extracellular chromatin structures released from PMNs present in dog sperm samples confirms the presence of NETs in all of them (Figure 1C, 2–8).

### 2.2. Visualization of the Different NET Phenotypes

**Figure 3:**
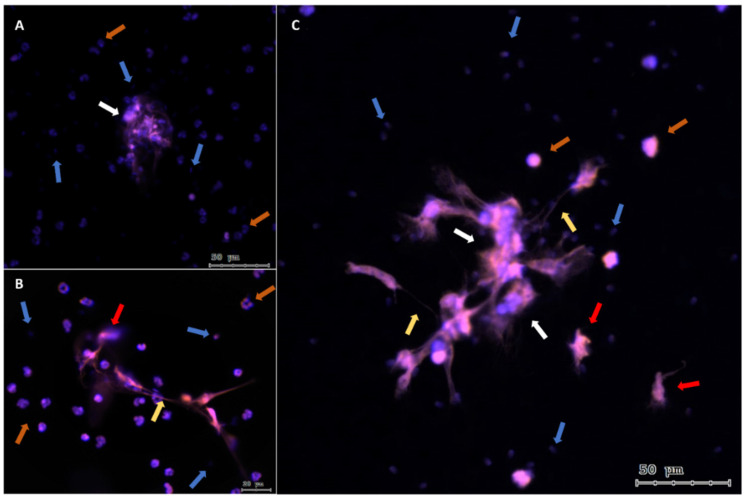
Representative immunofluorescence images of dog PMN/sperm co-cultures. (**A**–**C**) show the colocalization of the DAPI and Sytox Orange markers and different phenotypes of NETs. Orange arrows indicate PMNs, blue arrows indicate sperm, white arrows indicate *agg*NETs, yellow arrows indicate *spr*NETs, and red arrows indicate *diff*NETs ((**A**,**C**), scale bar = 50 μm, (**B**), scale bars = 20 μm).

**Figure 4:**
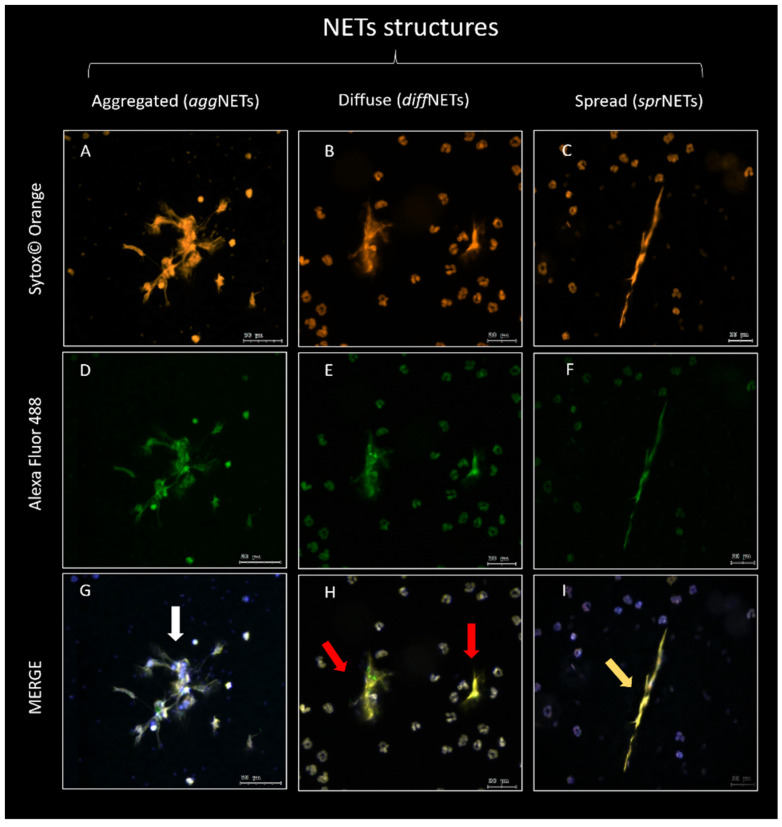
Representative images of the different NET phenotypes (*agg*NETs, *spr*NETs, and *diff*NETs) from PMNs stimulated with dog sperm. (**A**,**D**,**G**) The white arrow in (**G**) indicates the structure of *agg*NETs. In (**B**,**E**,**H**), the red arrows in (**H**) indicate *diff*NETs, and in (**C**,**F**,**I**), the yellow arrow in (**I**) indicates *spr*NETs. DNA can be observed with orange fluorescence in (**A**–**C**) (Sytox© Orange), and the presence of NE with green fluorescence (Alexa Fluor 488) in (**D**–**I**) shows co-location of both channels of extracellular structures attributable to NETs (scale bars = 20 μm).

Immunofluorescence analysis showed that PMNs from both canine seminal samples (Figure 2) and PMNs isolated from peripheral blood triggered the three different NET phenotypes currently described (*agg*NETs, *diff*NET, and *spr*NET) (Figure 3). Different-sized structures that trap the nearby sperm were observed; this can be seen through the co-location areas in these groups (MERGE; Figure 4G–I), with orange fluorescence (Sytox© Orange, DNA marker; Figure 4A–C), and green fluorescence (Alexa Fluor 488, NE marker; Figure 4D–F), which demonstrates an agreement in the marking attributable to the formation of NETs.

### 2.3. Quantification of NETotic Cells

**Figure 5:**
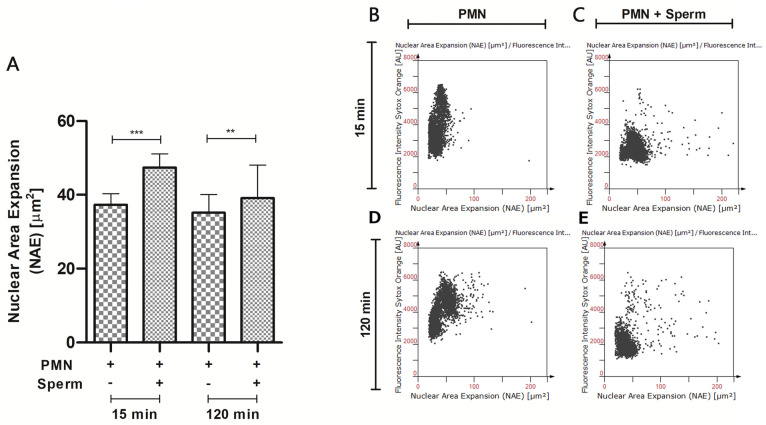
Representative image of nuclear area expansion (NAE) of PMNs isolated from peripheral blood exposed to dog sperm. (**A**) NAE−based quantification of sperm-activated PMNs at 15 and 120 min of exposure with their respective controls. The letters (**B**–**E**) represent scatter plots of PMNs nuclear segmentation. *p* < 0.01, **, *p* < 0.0001, ***.

The quantification of NAE indicated significant differences for the study groups at 15 min and 120 min compared to their control. A mean NAE expansion of 36.95 µm^2^ was observed for the 15 min control, while in the sperm-stimulated cells, the area increased significantly to 47.17 µm^2^ (*p* < 0.0001, ***). Similar results were observed at 120 min, where the mean NAE obtained from the PMNs group exposed to sperm showed significantly higher nuclear expansion of 39.14 µm^2^ (*p* < 0.01, **) compared to the control (35.18 µm^2^; Figure 5A).

### 2.4. Effect of MPO on Dog Sperm

**Figure 6:**
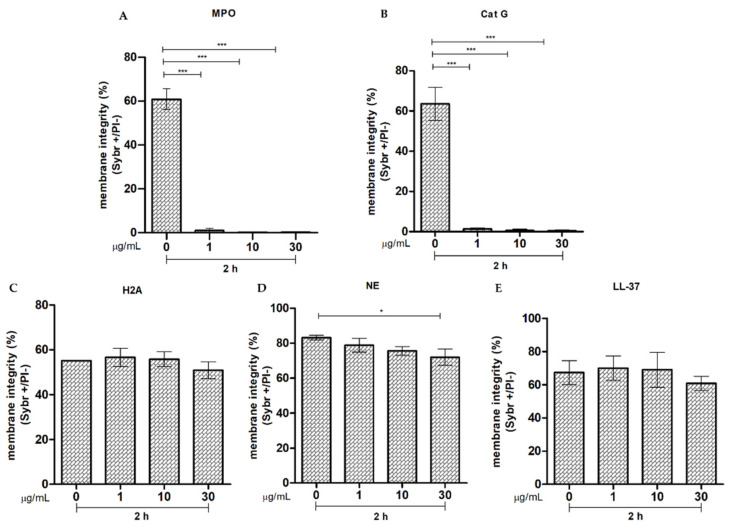
Effect of different concentrations of NET components on the cell membrane integrity of dog sperm. (**A**) Myeloperoxidase (MPO); (**B**) Cathepsin G (Cat G); (**C**) Histone 2 A (H2A), (**D**) Neutrophil elastase (NE), and (**E**) Cathelicidin (LL-37). The results are shown as mean ± standard deviation. The asterisks on the bar indicate differences in the three biological replicates compared to the control for MPO *p* < 0.02, *** (**A**); Cat-G *p* < 0.0001 *** (**B**), NE *p* < 0.05, * (**D**).

**Figure 7:**
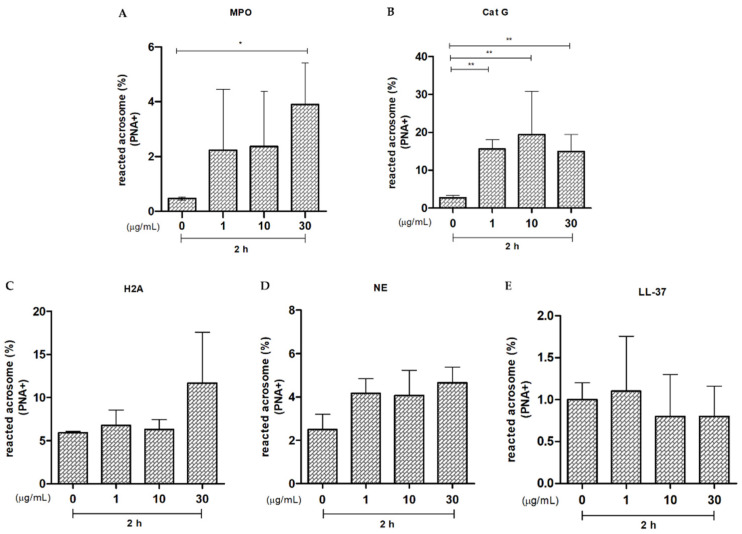
Effect of different concentrations of NET components on the state of the acrosome in dog sperm. (**A**) Myeloperoxidase (MPO); (**B**) Cathepsin G (Cat G); (**C**) Histone 2A (H2A), (**D**) Neutrophil elastase (NE), and (**E**) Cathelicidin (LL-37). The results are shown as mean ± standard deviation. The asterisks on the bar indicate differences in the three biological replicates compared to the control. For MPO *p* < 0.02, * (**A**); Cat-G *p* < 0.01 ** (**B**).

The parameter of membrane integrity after 2 h of exposure to MPO showed a significant reduction (*p* < 0.001, ***) in all experimental groups when compared to the control group (Figure 6A). The acrosome integrity showed that after 2 h of incubation of sperm with MPO, there was a tendency of reduced acrosome integrity when the protein concentration increased (1 µg/mL, 10 µg/mL, and 30 µg/mL) in a directly proportional way. However, there was only a significant difference (*p* < 0.02, *) in the study group with the highest protein concentration (30 µg/mL) compared to the control (Figure 7A).

### 2.5. Effect of Cathepsin G (Cat G) on Dog Sperm

The parameter of membrane integrity of the dog sperm after 2 h of exposure to Cat G showed a significant reduction (*p* < 0.0001, ***) in all experimental groups compared to the control (Figure 6B). The acrosome integrity showed that, after 2 h of incubation of sperm with Cat-G, there was a significant increase (*p* < 0.01, **) in the sperm population with acrosome disruption in all the study groups (i.e., 1 µg/mL, 10 µg/mL, and 30 µg/mL) (Figure 7B).

### 2.6. Effect of H2A on Dog Sperm

Sperm membrane and acrosome integrity showed no significant differences between the groups incubated for 2 h with different concentrations of H2A and the controls (Figure 6C and Figure 7C, respectively).

### 2.7. Effect of NE on Dog Sperm

There was a significant reduction in membrane integrity of the dog sperm after 2 h of incubation with NE only in the group exposed to 30 µg/mL compared to the control group (*p* < 0.05, *; Figure 6D). The results of the acrosome integrity test showed a tendency to lose integrity with the 30 µg/mL concentration. Nevertheless, there were no significant differences after 2 h of incubation with this protein (Figure 7D).

### 2.8. Effect of Cathelicidin (LL-37) on Dog Sperm

The studies for cathelicidin (LL-37) showed no significant damage to membrane or acrosome integrity in any of the study groups compared to the control (Figure 6E and Figure 7E).

## 3. Discussion

Sperm-mediated NET formation from seminal PMNs can occur in vitro and ex vivo in several mammalian species, including cattle, pigs, donkeys, and humans [18,19,20]. NETosis is an efficient defense process of the innate immune system and an essential mechanism to control bacteria, viruses, and parasites, but also to induce pathological inflammation and autoimmunity when released uncontrolled in tissues or vessels [9,11]. In addition to defensive properties, the impact of NETosis and METosis (extracellular traps released by monocytes) has been extended to the pathogenesis of several diseases (e.g., autoimmune diseases, coagulopathies, tumor progression) and also to the field of reproduction [20,21,22,23,24,25]. Physiologically, in mammalian reproduction, they are extruded to combat microbial pathogens and eliminate excess spermatozoa deposited after coitus within the FRT [26,27]. Nevertheless, the excess of PMNs in the FRT is detrimental because it triggers NETs against sperm, trapping and neutralizing them in these extracellular traps and altering their functional parameters. This may lead to the development of reproductive disorders as previously described in various mammalian species, including humans [19,20,28].

Our studies of semen samples from healthy dogs show the presence of abundant PMNs and NETs in the form of thin and thick chromatin fibers in the extracellular medium in all samples studied. The presence of NETs was observed not only in pathological samples but also in samples from healthy donors without associated infections [20], suggesting that physiological concentrations of basal NETs may be present in canine semen without causing alterations in semen parameters, or that released NETs are under control or adequately digested/removed by DNases also present in canine seminal plasma, as reported for other species [29]. Furthermore, it is known that sperm itself is a biological stimulus for the formation of NETs in vitro in different mammalian species [18,19,20,29] and, according to this finding, it could be expected that the PMNs present in canine semen follow the same activation promoted by the spermatozoon. In our in vitro model, canine sperm also strongly induced the release of NETs in PMNs, resulting in the rapid formation of *diff*NETs, *spr*NETs, and *agg*NETs, which efficiently trapped numerous sperm simultaneously, thereby neutralizing their progress. All three types of NETs result from numerous PMNs undergoing NETosis and producing a large number of elongated and/or diffuse fibers, with or without the formation of cell clusters that trap large numbers of motile sperm [18]. NETotic cells were also present in high numbers in PMNs and sperm co-cultures after a relatively short time. This result suggests that a significant number of PMNs are on the verge of releasing NETs, as NAE has been observed as a hallmark of early NETosis [30]. In this regard, very little is known about sperm-derived factors that promote NETotic cell induction and NET formation, while some studies suggest that it is the seminal plasma and not the sperm that triggers NETosis [31]. Conversely, other work suggests that it is an inherent property of sperm to induce this process, which is consistent with our observations [32,33]. The canine sperm in our model favors the formation of NETs, and it is still not entirely clear whether sperm motility would be a factor favoring PMN-derived NETosis. A recent study suggests that this phenomenon could be induced by sperm populations with high progressive motility, whereas dead and non-motile sperm populations could induce lower NETotic cell counts, resulting in lower NETs concentrations [33]. It is possible that mechanical movement stimulation can activate NETs from the PMN, a situation that also occurs with parasites [34,35].

The antimicrobial activity of NETs mainly affects pathogens that cause damage at the level of membrane integrity in bacteria, fungi, and parasites [21,26,36,37]. The results of this study demonstrated the individual damage induced by various NET proteins, including MPO, which is found in high concentrations in extracellular networks [9,38]. This membrane damage catalyzes the formation of a significant number of oxidative species [39], which can lead to further membrane lipid peroxidation in spermatozoa [32]. Similarly, our study demonstrated that MPO caused severe damage to the canine sperm membrane, where the acrosome was also affected. In addition, membrane damage in the presence of NET-derived MPO may be consistent with previous findings showing a progressive decrease in the motility of porcine sperm [19]. In addition, Cat G, one of the major serine proteases released by activated PMNs, is another protein associated with lipid peroxidation damage at the sperm level [32] and plays a critical role in inflammation through the hydrolysis of many other proteins [40]. Consequently, it was recently demonstrated that Cat G had detrimental effects on both sperm membrane and acrosome integrity in exposed bovine sperm [32]. NE has been involved in sperm-induced NET release in livestock such as cattle and pigs, which has also been shown to be associated with decreased motility and viability [32]. Furthermore, the presence of NE was associated with loss of sperm membrane functions, and these findings are consistent with our observations. NE-induced damage to membrane integrity suggests cytotoxic effects on canine spermatozoa which, in addition to the deleterious effects of other NET-associated proteins, may enhance sperm damage induced by the presence of extracellular NETs released by activated PMNs. These physiological responses of PMNs within the FRT after coitus will improve endometrial receptivity and embryo development prior to implantation. However, an increased or uncontrolled release of NETs resulting in pro-inflammation and tissue damage is an aspect to be considered, along with PMNs phagocytosis/degranulation, cytokine/chemokine production in studies of the uterine environment, and sperm progression in the canine reproductive tract.

## 4. Materials and Methods

### 4.1. Sample Acquisition and Ethical Declarations

The dog sperm samples were obtained from three healthy animals (using 3 semen samples per dog to carry out the experiments) of three breeds, i.e., a 3-year-old Chihuahua, a 2-year-old Cattle Dog, and a 3.5-year-old Dachshund. All protocols were approved by the Scientific Ethics Committee of the Universidad de La Frontera with the authorization code 120_20 and were conducted in compliance with Chilean Animal Protection Statute No. 20.380.

### 4.2. Place of Study

The experiments were conducted in the Sperm Biology and Conservation Laboratory CEMT-BIOREN at the Universidad de La Frontera, Temuco, Chile.

### 4.3. Reagents

All the reagents were acquired from Sigma-Aldrich (St. Louis, MO, USA), except where otherwise indicated.

### 4.4. Sperm Motility

The evaluation of total and progressive sperm motility was performed using the SCA^®^ computer system (software v.5.4.0.0) (Microptic S.L., Barcelona, Spain). A Motic-BA410 microscope (Xiamen, Fujian, China) was used equipped with a heating plate at 37 °C. Sperm samples (4 µL) were loaded onto a SCA^®^ Chambers slide and a minimum of 200 sperm were counted per measurement. 

### 4.5. Isolation of Canine PMNs

Blood sampling for the isolation of PMNs was performed using healthy dogs (n = 3) by cephalic vein puncture at the University Veterinary Clinic. Two mL of blood in EDTA tubes was dripped onto 2 mL of Histopaque 1077/Histopaque 1119 density gradient and centrifuged at 340× *g* for 30 min at room temperature (RT) in a swing rotor centrifuge, with no brake. The supernatant containing plasma and peripheral blood mononuclear cells (PBMC) was removed, and the PMNs sediment was carefully isolated by pipette aspiration. The sediment was washed thereafter with sterile Hank’s Balanced Salt Solution (HBSS) (Biochrom AG, Berlin, Germany), for 10 min at 300× *g*; the pellet obtained was resuspended in sterile lysis buffer and gently agitated for 10 min. Then, two washes were performed with a sterile HBSS medium, and the PMNs was resuspended in HBSS. Finally, the viability and purity of the PMNs were analyzed by exclusion with trypan blue (Sigma-Aldrich) in a Countess 3 FL system (Invitrogen, Thermo Fisher Scientific, Waltham, MA, USA).

### 4.6. May–Grünwald Giemsa Stain to Visualize PMNs

The smear was prepared with the native semen sample. The samples were covered with May–Grünwald solution for 5 min, the dye was eliminated, and the sample was covered with sterile PBS for 2 min. Then, the PBS was left to drain, and the sample was covered with diluted 5% Giemsa solution for 10 min. Finally, the sample was rinsed with potable water until the water ran clear. It was left to dry at RT and carefully covered with xylol–Entellan 1:2 [41].

### 4.7. PMNs Count by Microscopic Field Analysis of Seminal Smears

Quantification of PMNs occurred by the microscopic reading of smears of dog semen samples, expressed as leukocytes per field (LPF) using 40× magnification. Ten microscopic fields were read in homogenous zones of non-adjacent sites. The readings were performed with a magnification of 400× (fresh), with a cut-off value of 10 LPF, where the following parameters were considered: 0–10 scarce; 10–20 moderate; >20 abundant canine PMNs.

### 4.8. Analysis of NETotic Cells by Nuclear Area Expansion (NAE)

NAE analysis confirmed sperm-mediated NETosis induction by detecting NETotic cells as previously described, where the average of the nuclei was considered. Five 4 × 4 mm^2^ quadrants were randomly selected for each treatment, and Sytox© Orange fluorescence was measured. The PMNs nuclear areas of different NET-associated proteins in dog sperm were analyzed using StrataQuest v.7.0 software (TissueGnostics, Vienna, Austria).

### 4.9. Immunofluorescence for Neutrophil Elastase (NE) Detection

For the detection of NE, 200 µL of sperm sample per well were incubated for 15 and 60 min at RT. For a second experiment, the semen sample was washed at 300× *g* for 4 min, the semen plasma was removed, and the sperm free of semen plasma were resuspended in PBS. The sperm were co-incubated with PMNs in a 1:3 ratio for 15 and 120 min. The samples were fixed with 4% p-formaldehyde for 15 min, washed with sterile PBS, and blocked in PBS supplemented with 2% bovine serum albumin (PBS–BSA) for 30 min at RT. The samples were incubated overnight with a 1:300 dilution of anti-Rabbit IgG primary antibody (Abcam, Cambridge, UK) in PBS-BSA at RT. Then, the samples were washed three times with sterile PBS at 100 rpm agitation for 5 min and incubated for 1 h with a 1:500 dilution of anti-Rabbit IgG Alexa Fluor™ 488-conjugated secondary antibody (Invitrogen, ThermoFisher Scientific, Waltham, MA, USA) in 2% PBS-BSA at RT. The samples were then washed three times with PBS at 100 rpm in a classic orbital shaker (DLAB, Beijing, China) for 5 min and incubated with a 1:2000 dilution of SYTOX© Orange (Invitrogen) for 15 min at RT for DNA staining. Finally, the samples were washed with PBS and mounted with a mounting medium conjugated with DAPI (Invitrogen).

### 4.10. Exposure of Dog Sperm to Different NET Components

Three concentrations (1, 10, and 30 µg/mL) of five major NET components—histone 2A (H2A), myeloperoxidase (MPO), neutrophil elastase (NE), cathepsin G (Cat-G), and cathelicidin (LL-37)—were evaluated in relation to their effects on canine sperm. A sperm concentration of 2 × 10^6^ spermatozoa was used in 400 µL of PBS [32].

### 4.11. Sperm Membrane Integrity

The integrity of the sperm membrane was evaluated in a sperm suspension of 2 × 10^6^ in 400 µL of PBS incubated with the NET proteins described in Section 4.10. SYBR14^®^ fluorescent stains (final concentration, 1 nM) and propidium iodide (PI, final concentration, 5.8 µM) were used. The sperm were incubated for 10 min at 38 °C and then centrifuged at 300× *g* for 5 min. The sperm pellet was re-suspended in 200 µL of sterile PBS and analyzed thereafter by flow cytometry analysis.

### 4.12. Sperm Acrosome Integrity

Acrosome integrity was determined with peanut agglutinin (PNA) conjugated with fluorescein isothiocyanate (FITC)/propidium iodide (PI). PNA–FITC (final concentration 1 nM) and PI (final concentration 5.8 µM) were added to 400 µL of sperm suspended in PBS and incubated with NET proteins for 2 h. The sperm were incubated for 10 min at 38 °C and then centrifuged at 300× *g* for 5 min. The sperm pellet was re-suspended in 200 µL of PBS and then analyzed using flow cytometry. The percentage of negative populations was evaluated for PNA-FITC and PI (viable with intact acrosomes).

### 4.13. Flow Cytometry

The evaluation of sperm membrane and acrosome integrity parameters was conducted in a FACSCanto II flow cytometer (Becton, Dickinson and Company, BD Biosciences, San Jose, CA, USA). The samples were acquired and analyzed using FACSDiva^TM^ v. 6.1.3 (Becton, Dickinson and Company, BD Biosciences, San Jose, CA, USA). Ten thousand events per experimental group were quantified; manual selection was performed for the sperm population from the forward scatter channel (FSC) versus the side scatter channel (SSC).

### 4.14. Statistical Analysis

For the immunofluorescence experiments, one smear per sample obtained on different days with an n = 8 was evaluated. For flow cytometry studies, experiments were performed at least 3 times with different semen samples on different days. For both studies, results are presented as mean ± standard deviation (SD). The GraphPad Prism® software v. 9.4.1 was used for statistical analysis. The D’Agostino–Pearson K2 test was used to evaluate the Gaussian distribution; when the numerical results failed the normality test, they were transformed to a logarithmic scale. One-way analysis of variance (ANOVA) and Dunnett’s post-test were performed. Statistical significance was determined at *p* < 0.05. For the nuclear expansion statistic, the numerical results passed the normality test, then Student’s paired *t*-test was performed.

## 5. Conclusions

In conclusion, in this study we have for the first time demonstrated a significant increase in NETs extrusion in canine PMNs exposed to vital spermatozoa. The deleterious effects of these NETs on trapped spermatozoa were also demonstrated in terms of loss of membrane integrity as well as acrosome damage, expanding the knowledge of direct detrimental effects on male gamete. We established a correlation between the formation of NETs in co-cultures with motile sperm and the presence of different types of NETs (*diff*NETs, *spr*NETs, and *agg*NETs). The presence of PMNs and the possible formation of NETs may be a physiological response that aids in the elimination of bacteria, fungi, and parasites as well as excess spermatozoa. It will be essential to determine the role of sperm-mediated NETosis in vivo and to further clarify whether certain subpopulations of PMNs could be responsible for the generation of NETs at a level sufficient to induce a significant decrease in sperm number in situations of altered canine fertility.

## Data Availability

The data supporting the findings of this study are available from the corresponding author upon reasonable request.
